# Mature Cystic Teratoma at Fallopian Tubes: A Case Series

**DOI:** 10.31729/jnma.8149

**Published:** 2023-05-31

**Authors:** Suresh Kayastha, Akrity Bharati, Abhishek Shrestha, Swesha Shrestha, Asmita Pandey, Pratikshya Panday

**Affiliations:** 1 Grande City Hospital Pvt. Ltd., Kantipath, Kathmandu, Nepal; 2 Vatsalya Natural IVF, Grande City Hospital Pvt. Ltd., Kantipath, Kathmandu, Nepal

**Keywords:** *case reports*, *dermoid cyst*, *fallopian tube*, *infertility*

## Abstract

Benign tumors of the fallopian tube are uncommon. Teratomas are most frequently found in the ovary and fallopian tube teratoma is extremely rare. To date, around 70 cases have been described, and most of them were discovered by chance. Here we present two cases of fallopian tube dermoid cyst. The first case is of a woman who was unable to conceive for 4 years with a right ovarian dermoid. She was managed with laparoscopic cystectomy when she was found to have a small teratoma-like lesion at the fimbrial end of the left fallopian tube. The second case is of a female who underwent elective caesarian section and was found to have a teratoma-like lesion at the right fallopian tube. Histopathology of both cases were reported as mature cystic teratoma. These cases suggest the need for careful examination of the pelvic organs for other pathology apart from the primary surgical sites.

## INTRODUCTION

Mature cystic teratomas, also known as dermoid cysts, are made up of three germ layers: ectoderm, mesoderm, and endoderm. They develop from primordial germ cells. It is the most frequent benign ovarian tumor in women of reproductive age, accounting for 16 to 20% of all ovarian tumors.^1^ Benign fallopian tube primary tumors are far less common.^2^ Teratomas of the fallopian tube are extremely rare, with just about 70 cases recorded in the literature to date.^3^ Dermoid tumors of the fallopian tube are frequently discovered by chance following a cesarean birth or a diagnostic laparoscopy.^4^

## CASE 1

A 26-year-old nulligravida woman presented to the infertility clinic with complaints of inability to conceive for 4 years. There was no history of abdominal pain, vaginal bleeding, dysmenorrhoea, or dyspareunia. Her periods were regular and she denies any history of use of contractive in the past or any chronic illness. There was no prior surgical history. Gynecological examination was normal for her age.

**Figure 1:**
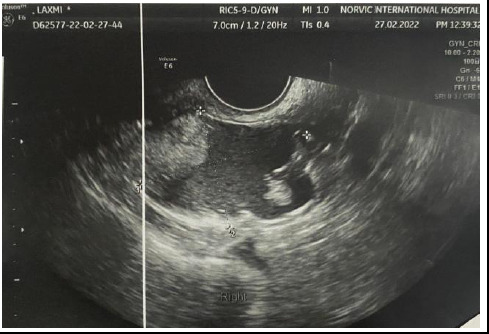
Ultrasonography showing cyst in right ovary measuring 50x35 mm with internal echoes and hyperechoic component, likely a dermoid cyst.

Her transabdominal ultrasonography revealed a cyst in the right ovary measuring 50x35 mm with a few dominant follicles in the left ovary largest measuring 11x11 mm (Figure 1).

**Figure 2:**
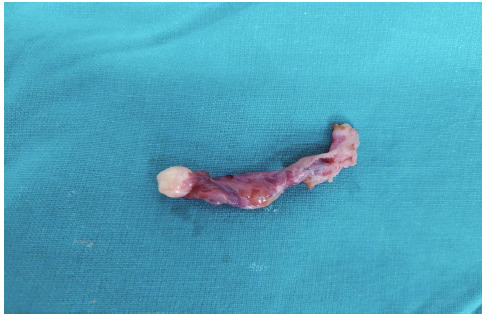
Post-operative image of left mature cystic teratoma of the fallopian tube

She underwent laparoscopic cystectomy for the right ovarian dermoid cyst. On chromopertubation, the left tube was blocked with a small teratoma-like growth of size 1x1 cm at the fimbrial end. After taking informed consent peroperatively, the right tube was seen to be patent and left salpingectomy was done (Figure 2).

**Figure 3:**
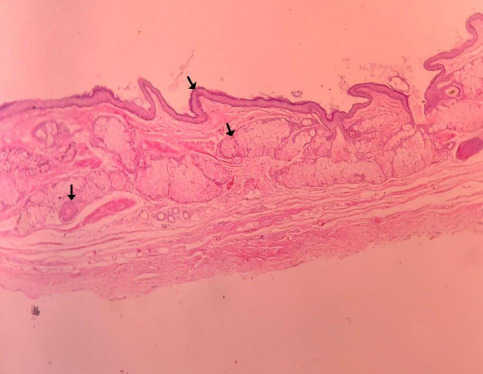
Histopathology image of left mature cystic teratoma of the fallopian tube showing laminated keratin, keratinized stratified squamous epithelium lying over the adnexal unit, composed of hair follicles, mature sebaceous glands.

She was discharged the next day after surgery. Her recovery was good. Later, histopathology of the left fallopian tube cyst showed laminated keratin, keratinized stratified epithelium lying over the adnexal unit, composed of hair follicles, and mature sebaceous glands, confirming the diagnosis of mature cystic teratoma of the left fallopian tube (Figure 3).

## CASE 2

**Figure 4:**
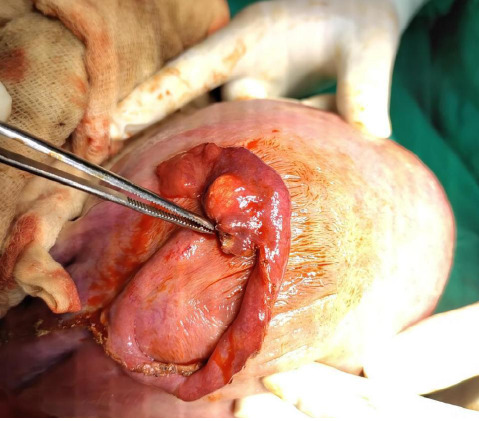
Small teratoma-like structure at the mesenteric border near the fimbrial end with the uterus exteriorized

42 years old female was admitted with the diagnosis of gravida 1 at 37 weeks of gestation, twin pregnancy with diamniotic dichorionic placenta following in vitro fertilization conception for unexplained subfertility for 16 years. Her prior fertility workups were normal, and she had a history of in vitro fertilization failure once before this pregnancy. Prior transvaginal or transabdominal scans did not reveal any suspicious lesions in the pelvis. She did not give a history of any past surgeries. She was planned for an elective caesarian section that was carried out under spinal anesthesia with the standard steps of the operating team. After suturing of the uterine incision, the uterus was exteriorized for examination of the pelvic structure, which revealed a small teratoma-like structure at the mesenteric border near the fimbrial end (Figure 4).

**Figure 5:**
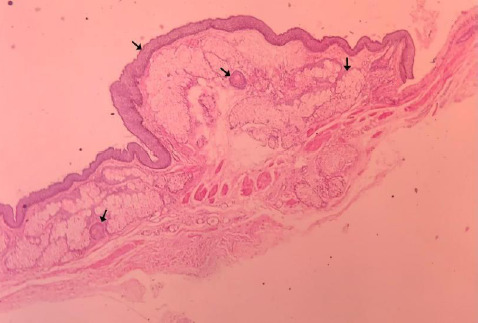
Histopathology image of right mature cystic teratoma of the fallopian tube showing laminated keratin, keratinized stratified squamous epithelium lying over the adnexal unit, composed of hair follicles, mature sebaceous glands.

Informed consent was taken, salpingectomy was done, and the specimen was sent for histopathological examination. She was discharged on day 3 of the postoperative period. Follow up histopathology report revealed laminated keratin, keratinized stratified squamous epithelium lying over the adnexal unit composed of hair follicles and mature sebaceous glands (Figure 5).

## DISCUSSION

Mature cystic teratomas are germ cell tumors that have well-differentiated tissues and three germ cell layers: ectoderm, mesoderm, and endoderm.^1^ These tumors are normally unilocular, but they can be multilocular and contain a variety of tissues in different ratios, including hair, skin, teeth, sebaceous material, cartilage, bone, salivary glands, and nerve tissue.^5^ Mature cystic teratomas are the most frequent kind of ovarian tumor, accounting for around 16-20 percent of all occurrences, with a peak frequency in women aged 20-40 years.^6^ Despite the fact that teratomas are the most common benign ovarian neoplasms, their occurrence in the fallopian tube is extremely rare. A survey of 73 tubal teratoma cases from throughout the world was published in 2013.^3^ Approximately 75 examples of tubal teratomas have been recorded in the English literature so far, with the majority of cases being cystic teratomas.^7^ Literature review does not show a similar case report from Nepal before.

Teratomas are thought to form from germ cells migrating from the yolk sac to the primitive gonadal bud. However, the pathophysiology of teratomas is unknown. Tubal teratomas may result from the failure of these germ cells to reach the ovaries.8 The majority of benign teratomas of the fallopian tube that occur in patients in their 40s are cystic and have significant size variations.^7^ The largest teratoma reported in the literature was approximately 30 cm in diameter and weighed 2400 gm.^9^ The majority of benign teratomas of the fallopian tube are unilateral and commonly affect one-third of the fallopian tube or the outer edge of the fallopian tube.^7^ In this case, the mature cystic teratoma of the left fallopian tube was associated with contralateral ovarian mature cystic teratoma in the first case, which is unusual because most mature cystic teratomas of the fallopian tube have been reported to be solitary.^1^

Teratomas are usually asymptomatic. Ovarian teratoma usually presents with abdominal pain, heaviness, or a mass. Less frequently, they can occasionally cause reduced parity or menstrual irregularity.^5^ It is obvious that a fallopian tube tumor drastically reduces fertility as it hampers the fertilization process. In each of the current cases, patients presented to the clinic with infertility and were managed with in vitro fertilization. In the first case, the left tube was found to be blocked and the right tube was patent during chromotubation while in the second case, hysterosalpingography was done and tubes were found to be patent. So, the reason for subfertility can not be attributed only to fallopian tubes and dermoids in both cases.

There is no test to confirm dermoids of fallopian tubes preoperatively. The laparoscopy or laparotomy and histopathology reports gives the definitive diagnosis. There have been no cases of preoperative diagnosis recorded to date. All of the documented cases were discovered incidentally during surgical procedures, and histopathological analysis later confirmed them. Because tubal teratomas are frequently misinterpreted as ovarian teratomas in radiologic examinations, pathologists must investigate the potential of a tubal teratoma when the origin of the adnexal mass is severely unclear.^5^

Teratoma is a rare neoplasm with the potential for malignancy. Therefore, early detection and excision of teratomas in any location are crucial.^10^ Following complete surgical excision, the prognosis is good.^11^

In conclusion, mature cystic teratomas of the fallopian tubes are uncommon. They are typically asymptomatic and discovered by chance. It is important to be aware of this possibility since radiologic investigations sometimes misdiagnose tubal teratomas as ovarian teratomas.
